# Computational and Experimental Approaches to Study the RNA Secondary Structures of RNA Viruses

**DOI:** 10.3390/v14081795

**Published:** 2022-08-16

**Authors:** Siwy Ling Yang, Riccardo Delli Ponti, Yue Wan, Roland G. Huber

**Affiliations:** 1 Genome Institute of Singapore, Agency for Science, Technology and Research (A*STAR), Singapore 138672, Singapore; 2 Bioinformatics Institute, Agency for Science, Technology and Research (A*STAR), Singapore 138671, Singapore

**Keywords:** RNA structure, RNA viruses, computational analysis, high throughput sequencing, structure modeling

## Abstract

Most pandemics of recent decades can be traced to RNA viruses, including HIV, SARS, influenza, dengue, Zika, and SARS-CoV-2. These RNA viruses impose considerable social and economic burdens on our society, resulting in a high number of deaths and high treatment costs. As these RNA viruses utilize an RNA genome, which is important for different stages of the viral life cycle, including replication, translation, and packaging, studying how the genome folds is important to understand virus function. In this review, we summarize recent advances in computational and high-throughput RNA structure-mapping approaches and their use in understanding structures within RNA virus genomes. In particular, we focus on the genome structures of the dengue, Zika, and SARS-CoV-2 viruses due to recent significant outbreaks of these viruses around the world.

## 1. Introduction

The recent COVID-19 pandemic once again highlights the social and economic impact of RNA viruses. RNA viruses contain an RNA genome that is translated into viral proteins and replicated to create more copies for packaging and growth. Important human RNA viruses include coronaviruses (e.g., SARS-CoV-2), flaviviruses (e.g., dengue and Zika viruses (DENV and ZIKV, respectively)), influenza viruses, human immunodeficiency virus (HIV), enteroviruses, and alphaviruses. Collectively, these viruses are responsible for sizable annual healthcare expenditure, loss of productivity, and, most significantly, lowered quality of life and increased mortality [1,2,3]. Understanding the key mechanisms of these RNA viruses in terms of how they function and how these mechanisms can be targeted is crucial to our ability to control, manage, and treat these viruses and their diseases.

Traditionally, most therapeutics against RNA viruses target either the viral proteins or key host receptors/host interacting proteins inside cells [4,5]. These strategies can be limited by the availability of binding pockets on viral and host proteins, off-target effects of drugging host proteins, and the ability to identify viral receptor proteins on host cells. In recent years, studies have revealed that the information required for viral replication and pathogenicity is not only encoded in their linear RNA genomes but also in the ability of the genomes to form complex higher-order structures. The genomes of RNA viruses can fold into secondary and tertiary structures to regulate almost every step of the viral life cycle [6]. This complexity of viral RNA structures makes it potentially feasible to develop small-molecule compounds to disrupt them, thus creating an entirely new class of drug targets [7].

In this review, we summarize recent efforts in understanding the structure and function of RNA viruses, with a focus on DENV/ZIKV and SARS-CoV-2, as these were involved in recent pandemics. DENV is responsible for approximately 390 million infections (of which 100 million are symptomatic) and 36,000 deaths per year, with approximately 70% of the disease burden residing in Asia [1]. While most patients recover following non-severe disease, a small proportion progress to severe disease, characterized by plasma leakage with or without hemorrhage, potentially resulting in fatal dengue shock syndrome [8]. The average illness duration is approximately 12 days, and treatment costs have been estimated at 514 international dollars (I$) for ambulatory patients and I$1394 per hospitalized patient in 2009, with recent estimates at 1472 USD (+/− 1695 USD) for ambulatory cases and 3416 USD (+/− 2188 USD) for hospitalised cases [9].

As the SARS-CoV-2 pandemic is still in progress, estimations of the total burden of SARS-CoV-2 are by necessity preliminary and subject to later revision. As of July 2022, there have been over 500 million documented SARS-CoV-2 infections resulting in over 6 million deaths [10]. In addition to the loss of life, a significant number of patients are suffering from after-effects of severe infections (long-COVID) [11]. Governments around the world have implemented disease control measures that have led to widespread economic disruptions, particularly in supply chains and international trade, persisting to this day. Healthcare systems around the world have been operating at or beyond the limit to deal with the COVID-19 pandemic, resulting in reduced capacity for non-COVID-related patients [12].

Here, we describe advances in computational and experimental RNA structure-mapping methods that have greatly facilitated our ability to identify new, functionally important RNA structures along viral genomes. We also discuss potential gaps that need to be filled for viral RNA to be made into viable drug targets.

## 2. Existing Computational and Experimental Approaches to Mapping Viral RNA Structures

While RNA structures have long been known to be functionally important in viral genomes, much of what we previously knew about RNA structures was serendipitous. Such functional structures include the internal ribosomal entry site (IRES) on hepatitis C virus (HCV), enteroviruses, and polioviruses [13,14,15,16]; pseudoknot elements that facilitate ribosomal frameshifting to control viral protein production in HIV and coronaviruses [17,18]; and structures in 5′ and 3′ untranslated regions (UTRs) that can recruit RNA-dependent RNA polymerase for genome replication in DENV and ZIKV [19]. Due to the traditional challenges of obtaining RNA structures using X-ray crystallography, nuclear magnetic resonance, and cryo-electron microscopy (cryo-EM), the full extent and distribution of RNA structural elements and their functions along virus genomes remained largely unknown.

## 3. Computational Approaches to Studying RNA Virus Genome Structures

The difficulty of determining RNA structures experimentally prompted the widespread use of computational predictions using sequence information. These include thermodynamics-, simulation-, and AI-based approaches to predicting RNA structure (Table 1). The most frequently used computational approaches for predicting RNA secondary structure are thermodynamics-based folding algorithms, including RNAstructure [20] and RNAfold [21]. Their main advantage is that they can predict RNA secondary structures using only the sequence of interest and without requiring any experimental data or complementary information. These algorithms sample every structure that can be obtained from the RNA sequence by following a set of folding rules (i.e., nucleotides allowed to pair) and search for the most probable native structure; i.e., the conformation with the minimum free energy (MFE). To compute the free energy of an RNA secondary structure, thermodynamics-based algorithms use a set of parameters that were first determined by optical melting experiments [22]. However, thermodynamics-based dynamic programming methods are heavily limited by the length of the RNA sequence. They are accurate for shorter sequences, but performance drops drastically for those longer than 700 nucleotides [23,24]. This limits their utility for long and complex RNAs, such as single-stranded RNA (ssRNA) viral genomes. Nevertheless, these algorithms continue to be widely used on viral genomes, generally with a locality constraint, mostly for lack of alternatives. Recent improvements that allow the integration of experimental constraints to guide RNA structure prediction have been shown to improve modeling accuracy [25].

When an RNA sequence contains many known homologs or shows strong structural conservation [26], information from homologous sequences can be exploited to build algorithms with high predictive accuracy. Some of these tools, such as RNAalifold [27] and TurboFold [28], work by extracting information from multiple sequence alignments, which are then used to predict the secondary structure. The main difference between the two approaches is that while RNAalifold uses the obtained consensus sequence to predict the structure, TurboFold individually computes all the structures inside the alignment. Other algorithms, such as Dynalign/Multilign [29,30] and FoldalignM [31], generate the alignment and predict the structure at the same time, making them more broadly applicable but also more computationally expensive (Table 1). Algorithms based on comparative sequence analysis are well-suited to studying viral RNA genomes for which multiple strains and phylogenomic data can be employed to support structural evidence. This is possible due to well-curated online repositories of complete viral genomes. For example, the Virus Pathogen Resource (https://www.viprbrc.org) (accessed on 23 May 2022) [32] is currently hosting thousands of complete genomes for different emerging viruses, including >7000 DENV, >1000 ZIKV, and >750 Ebola virus (EBOV) genomes, as of May 2022. The dataset is also an important repository during the COVID-19 pandemic and includes more than 4 million SARS-CoV-2 genomes. Other specialized datasets include multiple genomes of a single virus, such as hivdb (https://hivdb.stanford.edu/) (accessed on 23 May 2022), which contains >20,000 HIV genomes.

The information from viral sequences can also be used to study mutation landscapes. When a mutation happens on a base that is paired and the pairing is functionally important, there will typically be evolutionary pressure to incur additional mutations that restore the base-pairing over time. The existence of such mutations is called covariation. Multiple sequence alignments are used to compute sequence covariation at a single-nucleotide resolution to identify these covaried and presumably functionally important structures. This multiple sequence alignment is used by algorithms such as R-scape to support the presence of conserved RNA secondary structure elements [33]. In DENV and ZIKV, Huber et al. used information from >4000 DENV (DENV1–4) and >500 ZIKV sequences to build covariation profiles to analyze regions with low synonymous mutation rates and low SHAPE reactivity (highly structured regions, as described in the next section) and characterize structural similarity between the two viruses [34]. 

## 4. Experimental Approaches to Studying RNA Virus Genome Structures

The difficulty in obtaining structural information for long RNA sequences was considerably reduced in 2009 when the entire HIV-1 genome was structurally mapped experimentally by SHAPE chemical probing [35]. Secondary structure probing along the HIV-1 genome was performed using SHAPE (1M7), with the resulting RNA 2′OH acylation identified by primer extension using fluorescently labeled primers and capillary electrophoresis across the entire genome. Watts et al. identified relationships between RNA structure and protein domains, suggesting that increased structuredness could slow down translation to allow protein domains to fold properly [35]. Since then, other studies have coupled biochemical approaches to high-throughput sequencing to increase our ability to obtain large-scale RNA structure information. These new methods, including SHAPE-MaP [25], icSHAPE [36], DMS-MaPseq [37], SPLASH [38], PARIS [39], COMRADES [40], and vRIC-seq [41] (Table 2), revolutionized our ability to obtain RNA structure information along viral genomes and families of viral genomes at high speed. With these technologies, we are able to determine paired and unpaired bases [34], as well as their base-pair partners along the genome, enabling us to dissect the global map of viral RNA structures in vitro, in virions, and inside the host cells. Over the years, structural studies have been performed on different RNA virus genomes, including the poliovirus, simian immunodeficiency virus (SIV), HCV, DENV, ZIKV, influenza A virus (IAV), and SARS-CoV-2 (Table 3). These studies have demonstrated that structured RNA elements are pervasive across viral genomes, can be remodeled, and are involved in diverse biological processes. By combining studies of structure with those of mutagenesis and function, we and others have shown that RNA structures are associated with viral functions in replication, protein translation, packaging, evasion of host immune response, and hijacking of host cell machinery. These findings reveal the sophisticated nature of RNA structure–function relationships and provide potential approaches for targeted therapy.

## 5. Combining Experimental and Computational Approaches to Identify Functional RNA Structures

Data from a high-throughput RNA structure-probing experiment using SHAPE or dimethyl sulphate (DMS) can be in the form of either the number of reverse transcriptase (RT) drop-offs or the frequency of mutation errors along each base of an RNA [42]. Counting the number of RT drop-offs is the foundation in methods such as icSHAPE [36], while counting mutational errors forms the basis of mutational mapping (MaP) methods, such as SHAPE-MaP and DMS-MaPseq [25,43]. The increasing mutational frequency of a base in the structure-probed sample increases the likelihood that the base is single-stranded, as flexible bases tend to react more readily with the structure-probing compounds. This information can be integrated into RNA structure modeling to generate more accurate RNA structure predictions (Box 1) across an entire genome. Additionally, to determine the sites of base pairing along the genome, we can identify the base-pairing regions by proximity ligation sequencing. Several methods, including SPLASH [38], PARIS [39], COMRADES [40], LIGR-Seq [44], and RIC-seq [45], have been developed to identify pairwise RNA interactions, with the information obtained visualized using a two-dimensional RNA contact map. This information can also serve as constraints in structural models to generate more accurate secondary and tertiary structure models [34]. However, identifying functionally relevant structures amongst these candidates remains a challenge.

To identify functional structures along viral genomes, regions that are highly structured (low SHAPE reactivity) and contain low Shannon entropy [34,46] (which indicates the likelihood of elements forming unique structures) have been nominated. Another common algorithm used to analyze RNA structural stability is the program ScanFold [47]. ScanFold assesses the stability of secondary structure elements in a region-size of interest by ranking regions that fold into more stable conformations than can be expected to occur by random chance [48,49]. Additionally, we can use evolutionary information to further refine functional RNA structures. For example, the algorithm RNA-Decoder [50] profiled the complete HIV-1 genome by examining the evolutionary information contained in its nucleotide and amino acid variation and assigning pairing probabilities at the single-nucleotide level [35]. The algorithm uses a set of grammar parameters, a multiple-sequence alignment, and a phylogenetic tree as inputs to provide pairing probabilities for each nucleotide in the HIV genome. As such, SHAPE reactivity can be used in combination with Shannon entropy, ScanFold, and RNA-Decoder to increase our confidence in identifying functional structures (Table 1). Using a combination of these strategies, scientists have identified numerous potentially functional structural elements along RNA virus genomes, including known HIV regulatory elements, such as the 5′ UTR, the Rev responsive element (RRE), and known DENV and ZIKV regulatory elements. Recently, these strategies have been automated using the pipeline RNAvigator [51], which uses these data to correctly predict different viral elements, including the 3′ UTR of DENV-1 and the IRES element of HCV. Moreover, a similar approach was also used for SARS-CoV-2 to identify >20 regions that could be used for therapeutic siRNA targeting [49].

With increased structural mapping of RNA viruses with SHAPE-MaP and DMS-MaPseq, single-molecule analysis of the mutational profile of each read along the virus genome has shown that viral RNA structures are more complex than previously thought and can take on alternative structures. Although alternative structures have been observed from proximity ligation sequencing experiments [34], as much as 50% of the SARS-CoV-2 genome and 90% of the HIV genome have been observed to form two or more structures [43,52] in studies using single-molecule clustering. These alternative structures are functionally significant and can impact splicing rates in HIV and ribosome frameshifting efficiencies in SARS-CoV-2. The elucidation of functional genomic structures in RNA viruses further facilitates our ability to target them.

Box 1 **Combining experimental and computational approaches for structure modelling of RNA.**

High-throughput experimental techniques such as SHAPE are able to profile the RNA at single-nucleotide resolution. However, they can only provide a reactivity that associates each nucleotide with the propensity to be in single- or double-stranded conformation. It has recently been shown that experimental data can be used as soft constraints inside thermodynamics-based algorithms [53,54]. Another such experimental technique is Superfold, a folding algorithm originally developed to work by using SHAPE data and created by the same group behind SHAPE [25].

While hard constraints force the algorithms to work through rigidly-imposed structures, soft constraints guide the algorithms by providing more degrees of freedom during folding. Hard constraints assign a specific conformation to a nucleotide and thus reduce the degrees of freedom of the search. On the other hand, soft constraints work as guidelines, using the experiments as a continuous signal, where a weak signal allows more degrees of freedom to the algorithm without imposing a structure. Due to their flexibility, soft constraints are widely used and accepted by the majority of the algorithms. RNAstructure and RNAfold both accept DMS and SHAPE data as soft constraints [23,53]. Especially SHAPE data were successfully integrated into thermodynamic algorithms, providing not only the visualization of the obtained structure but improving the performance of the algorithms considerably. Incorporating experimental data as soft constraints improved prediction accuracy up to 90% [23,54].

To work as soft constraints, SHAPE reactivities are converted into pseudo-energy contributions [55]. A common approach for this transformation is to convert the reactivities for each nucleotide into the probability of being unpaired [56]. These unpairing probabilities are then used to compute two pseudo-energy weights for each nucleotide, considering the nuleotide in both the possible paired or unpaired conformations. Another strategy considers the pseudo-energy conversion as an optimization problem, using a perturbation vector to find the minimizing energies [57]. The folding algorithms then use the converted pseudo-free energies to assign bonuses or penalties when the calculations are in agreement with experimental data.

The integration of experimental profiles and predictive algorithms was successfully applied to almost every model of secondary structure for ssRNA viruses, including HIV, ZIKV, DENV, and SARS-CoV-2 [34,35,49]. Moreover, recent AI-based algorithms were trained on SHAPE data [58,59], showing good results when predicting the secondary structure of viral genomes, such as those of SARS-CoV-2 and DENV [60,61].

## 6. The Structurome of RNA Viruses Involved in Pandemics

While the structures of RNA viruses have been studied in the past decade, the need to understand RNA virus structure and function has been recently heightened due to two pandemics (Table 3). Understanding how the viral genome folds and interacts with viral and host factors can help in identifying potential targets against these viruses. Here, we focus our attention on structural studies of DENV/ZIKV (the latter responsible for the 2015–16 Zika epidemic) and SARS-CoV-2 (responsible for the ongoing COVID-19 pandemic starting November 2019).

Dengue/Zika viruses belong to the Flaviviridae family of viruses, which includes the West Nile, HCV, and yellow fever viruses, all of which are clinically important to our society. Prior to the 2015 Zika outbreak, most attention had been focused on DENV, its close relative. DENV infects >390 million people around the world each year, imposing a high social and economic burden [1]. Presentation of dengue infection ranges from a mild fever to potentially fatal hemorrhagic fever and the associated dengue shock syndrome. The 2015 ZIKV epidemic originated in Brazil and quickly spread to other parts of the world, ZIKV was thought to result in microcephaly in newborns and other neurological diseases [62]. Although DENV and ZIKV only share about 60% of their sequence identities, many of their key structural features are conserved. These structures include elements in the 5′ and 3′ UTRs, which are known to be important for viral replication and translation and can pair with each other to facilitate genome circularization [63]. However, prior to the epidemic, other structural elements along the virus genome were not fully understood or studied.

**Figure 1:**
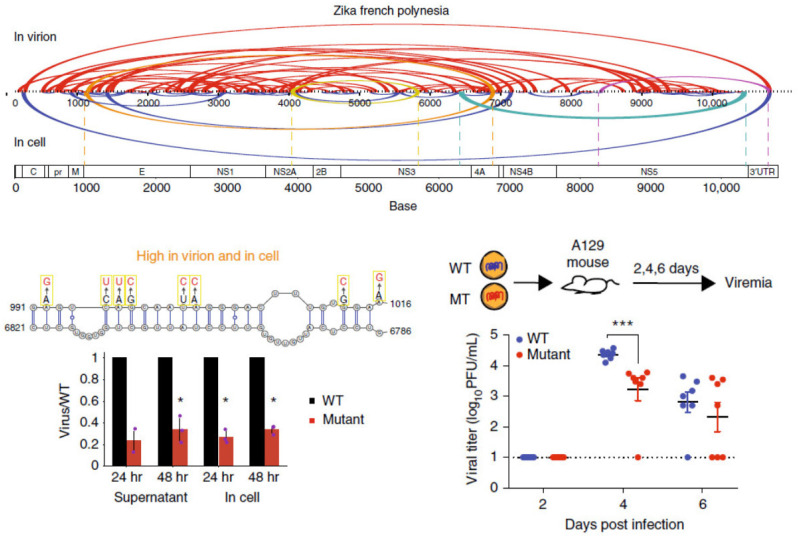
(**Top**), pairwise RNA–RNA interactions along the Zika virus genome when the genome is inside virion particles and inside cells. (**Bottom left**), RNA cofold models of pairwise RNA interactions and mutations along the interaction. Mutations decrease the ability of the virus to grow inside cells. * *p* < 0.05 (Student T-test, two-tailed). (**Bottom right**), mutant viruses show lower levels of viremia in mice, indicating that they are attenuated. *** *p* < 0.001 (Mann-Whitney U test). Image retrieved from an open access article [34] distributed under the terms of the Creative Commons CC BY license.

Using a combination of high-throughput structure-probing strategies, the genome organizations of DENV and ZIKV in virions and inside infected cells were probed, and it was observed that many genome interactions are associated with virus growth (Figure 1). Using SPLASH and SHAPE-MaP, we observed that their genomes are structurally heterogeneous, although structure probing across different DENV and ZIKV serotypes revealed a generally conserved macro-organization of the RNA genome inside virions [34]. Using PARIS and icSHAPE, Zhang et al. identified long-range functional RNA structures unique to the Asian-specific lineages in ZIKV [64]. In addition to identifying secondary structures, Week et al. also identified potential tertiary structures that could play important roles in virus fitness using RING-Map [65]. Collectively, these findings serve as a resource for designing therapeutics to target genomic structures in DENV and ZIKV.

The COVID-19 pandemic has brought our attention back to the coronaviruses. Following the SARS outbreak in Hong Kong in 2002–2004, there has been a coronavirus outbreak about every decade. With a genome size of 26–32 kb, coronaviruses are some of the largest RNA viruses that exist [66]. RNA structures in the 5′ and 3′ UTRs, and particularly in the frameshifting elements, are known to be important for virus function. However, the way in which the rest of the genome is folded, and the functions of structures within the genome, remain largely unknown. Several groups in the RNA structural biology field have been actively investigating the structure of the SARS-CoV-2 genome using different probing strategies since the COVID-19 outbreak (Table 3).

**Figure 2:**
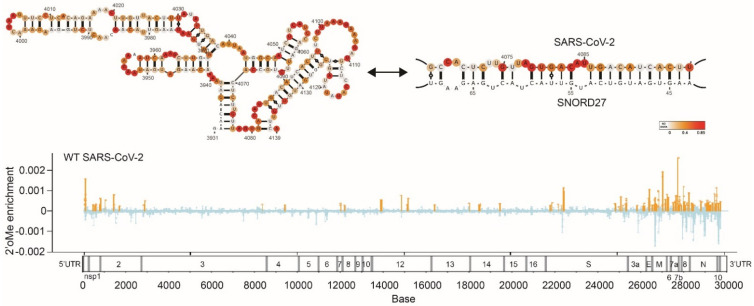
(**Top**), SARS-CoV-2 structure models when the SARS-CoV-2 genome does not interact or interacts with SNORD27. SHAPE reactivity was used for constraints in this model. (**Bottom**), locations of 2′-O-methylation sites found along SARS-CoV-2 genome. Image retrieved from an open access article [49] distributed under the terms of the Creative Commons CC BY license.

These comprehensive structural analyses reveal different aspects of the SARS-CoV-2 genome and biology, including its ability to form many short- and long-range RNA interactions, higher-order RNA conformations, and alternative structures. The SARS-CoV-2 genome also interacts extensively with host RNA-binding proteins for infectivity [67,68]. Additionally, Lan et al. observed that the frameshifting element can take on alternative functional structures and that its ability to fold into a functional element requires long-range RNA interactions that span >1 kb [52]. We also observed that the SARS-CoV-2 genome can interact with SNORD27 and can be 2′-O-methylated to increase its own genome stability, suggesting that hijacking the hosts’ cellular machinery can promote virus survival (Figure 2) [49]. Lastly, identifyiing long-range RNA interactions have also enabled the three-dimensional genome inside virion particles to be modeled [41]. Cao et al. observed that different regions of the genome occupy different territories inside the virion particle, similar to the 3D architecture of the genome in human cells. Understanding the genome structure of SARS-CoV-2 facilitates the identification of siRNAs, ASOs, small molecules, and other drugs that can target the genome [68,69]. For example, small molecules coupled with degraders (RIBOTAC) have been identified that can work against the SARS-CoV-2 genome [69]. Additionally, 69 small molecules have also been found to bind to the SARS-CoV-2 genome [70]. Collectively, these studies enable us to better understand the coronavirus genomes and to develop new therapies for SARS-CoV-2 and other coronaviruses.

## 7. Discussion

Recent advances in computational modeling and high-throughput experimental structure-probing have greatly increased our understanding of the diversity and prevalence of structures along RNA virus genomes. In combination with biochemical probing, high-throughput sequencing, and structural modeling, we can now map RNA structures along the viral genome, visualize what they potentially look like, and identify potential functional structures that are conserved through evolution. While much of the structural information exists as an aggregate across all copies of the detected RNA genomes, recent developments in single-molecule structure clustering have increased our resolution of RNA structures by identifying alternative structures along the genome. Used together with mutagenesis and functional assays, we can now identify RNA structures that play important roles during the virus lifecycle.

While current technologies provide a snapshot of what the RNA virus genome looks like at a given point in time, viral genomes are constantly going through different stages of the viral lifecycle, such as replication, translation, and packaging. As such, structure mapping of viral genomes can only provide an aggregate picture of structures across all stages of its lifecycle. Further studies on viral genome conformations at different stages of the virus lifecycle will facilitate our understanding of RNA structures that are specific to viral replication, translation, and packaging. Additional information on the location of heterogenous RNA structures, such as in HIV-1 and SARS-CoV-2, and, in particular, the identification of the functional alternative structures, will greatly facilitate our understanding of RNA structure and function [43,52,71].

Through comprehensive structure probing of virus genomes, we can now identify potential functional structures for therapeutic targeting by small RNAs (including siRNAs and antisense oligos) which preferentially target single-stranded regions along the virus genomes. Additionally, recent developments in RNA drug targeting have demonstrated RNA structures to be effectively targeted by small molecules due to their ability to bind to compounds with high specificity and affinity [7]. As such, experimental and computational screens have been developed to identify small molecules that can bind to RNA structures of interest [69,70,72]. One recent advance that has been key in facilitating small-molecule RNA drug development is the development of more rapid Cryo-EM technologies, which allow us to obtain RNA structural information at near-atomic resolution [73]. While there are currently very few high-resolution viral structures identified (e.g., frameshifting element of SARS-CoV-2 [74] and stem-loop A of DENV [75]), we anticipate the identification of more high-resolution structures in the near future. These will provide the structural information needed for molecular docking and in silico screens and enable the refinement of potential lead compounds that bind to the RNA structure. Collectively, our ability to elucidate RNA structures enables us to better understand the structure–function relationship of RNA viruses and allows us to better target them.

**Table 1:** Algorithms to predict and study RNA secondary structure.

Application	Methods	Algorithm Purpose	Input	References
Prediction of RNA secondary structure				
Thermodynamics-based	RNAfold, RNAstructure	Predicts the RNA secondary structure of a standalone sequence	RNA sequence	[20,21]
Comparative-based	RNAalifold, TurboFold, Dynalign, Multilign, FoldalignM	Predicts the RNA secondary structure using multiple sequences	Multiple-alignment/RNA sequence	[27,28,29,30,31]
AI-based	CROSS, ShaKer	Predicts the RNA secondary structure of a standalone sequence	RNA sequence	[58,59]
Combined approaches	RNAfold, RNAstructure, Superfold	Predicts the RNA secondary structure using experimental data as constraints	RNA sequence, SHAPE profile	[20,21,25]
Identification of functional RNA structures				
Secondary structure conservation	R-scape, RNA-Decoder	Identify covariation, base-pairing probability across many sequences	Multiple-alignment	[33,50]
Stability and potential functionality	ScanFold, RNAvigator	Identify RNA regions that are more experimentally stable than expected, identify regions of structural importance	RNA sequence, SHAPE profile	[47,51]

**Table 2:** High-throughput global mapping strategies for RNA secondary structures and tertiary conformations.

Method	Chemical Probe	Strategies	Advantages	Limitations	References
SHAPE-MaP	1M7, NAI, 2A3	Use SHAPE compounds to probe ssRNA regions. The mutations are detected through RT mutation read throughs	Probes all four nucleotides, analysis of low-abundance RNAs	Low jump through mutation rate, requires deep sequencing, no dsRNA information	[25,46,49,76,77]
icSHAPE	NAI-N3	Probes ssRNA regions, biotin enrichment for modified fragments, RT-stop read out	Probes all four nucleotides, high signal-to-noise ratio	No dsRNA information	[36,64,78]
PARIS	AMT crosslinking	Psoralen-based crosslinking of dsRNAs, 2D gel extraction, proximity ligation and sequencing	Genome-wide in vivo RNA–RNA interactions, near base-pair resolution	Psoralen preferentially integrates into pyrimidine-rich sequences, proximity ligation in dilute solution	[39,64]
COMRADES	Psoralen-TEG-azide crosslinking	Psoralen-based crosslinking of dsRNAs, enrichment of RNA of interest using biotinylated probe, second biotin enrichment for crosslinked regions, proximity ligation and sequencing	Genome-wide in vivo RNA–RNA interactions of a specific RNA	Psoralen preferentially integrates into pyrimidine-rich sequence, proximity ligation in dilute solution	[40,79]
SPLASH	Biotinylated-psoralen	Psoralen-based crosslinking of dsRNAs, biotin enrichment for crosslinked regions, proximity ligation and sequencing	Genome-wide in vivo RNA–RNA interactions, high signal-to-noise ratio	Psoralen preferentially integrates into pyrimidine-rich sequence, proximity ligation in dilute solution	[34,38,49,80,81]
RING-MaP	DMS	DMS methylation on A and C, RT mutation read out	Structure probing of RNAs in 3D tertiary conformations	Only probes As and Cs, requires deep sequencing, and is mostly used for highly abundant RNAs	[65,82]
DMS-MaPseq	DMS	DMS methylation on A and C, RT mutation read out	Structure probing of RNAs in multiple conformations, analysis of low-abundance RNAs, high signal-to-noise ratio	Only probes As and Cs, no dsRNA information	[37,52,76]
PORE-cupine	NAI	Probes ssRNA regions, RT mutation read out using Nanopore full-length direct RNA sequencing	Probes all four nucleotides. Long-read sequencing enables capture of structural information of RNA isoforms and full-length transcripts	No dsRNA information, low sequencing depth.	[49,83]
vRIC-Seq	Formaldehyde crosslinking	In situ RNA digestion by nuclease, in situ proximity ligation, biotin enrichment for ligated fragments	Genome-wide in vivo RNA–RNA interactions, high signal-to-noise ratio, high percentage of chimeric reads	Formaldehyde crosslinking may introduce protein–protein, along with protein–RNA, interactions	[41,45]

**Table 3:** Genome-wide RNA structure studies in viruses.

Virus Family/Genus	Virus Species	Methods	Year	Reference
Retroviridae/Lentivirus	HIV-1	SHAPE	2009	[35]
	SIVmac239, HIV-1	SHAPE	2013	[84]
	HIV-1	SHAPE-MaP	2014	[25]
	SIVcpz, SIVmac, HIV-1	SHAPE	2015	[85]
Picornaviridae/Enterovirus	Poliovirus	SHAPE	2013	[86]
Flaviviridae/Hepacivirus	HCV	SHAPE-MaP	2015	[46]
	HCV	SHAPE	2016	[87]
Flaviviridae/Flavivirus	DENV2	SHAPE-MaP, RING-MaP	2018	[65]
	ZIKV	icSHAPE, PARIS	2018	[64]
	ZIKV	COMRADES	2018	[40]
	DENV1–4, ZIKV	NAI-MaP, SPLASH	2019	[34]
Orthomyxoviridae/ Alphainfluenzavirus	IAV	SHAPE-MaP, SPLASH	2019	[80]
	IAV	2CIMPL	2021	[88]
Coronaviridae/Betacoronavirus	SARS-CoV-2	Nanopore DRS, DNBseq	2020	[89]
	SARS-CoV-2	SHAPE-MaP, DMS-MaPseq	2020	[76]
	SARS-CoV-2	COMRADES	2020	[79]
	SARS-CoV-2	icSHAPE	2021	[68]
	SARS-CoV-2	SHAPE-MaP	2021	[48]
	SARS-CoV-2	vRIC-seq	2021	[41]
	SARS-CoV-2	SHAPE-MaP, PORE-cupine, SPLASH	2021	[49]
	SARS-CoV-2	Simplified SPLASH	2021	[81]
	SARS-CoV-2	DMS-MaPseq	2022	[52]
	SARS-CoV, MERS-CoV, SARS-CoV-2	SHAPE-MaP	2020	[90]

## Data Availability

Not applicable.
